# Path-Based Discrete Modeling and Process Simulation for Thermoplastic Fused Deposition Modeling Technology

**DOI:** 10.3390/polym17081026

**Published:** 2025-04-10

**Authors:** Zhuoran Yang, Feibo Wang, Yiheng Dun, Dinghe Li

**Affiliations:** 1 Sino-European Institute of Aviation Engineering, Civil Aviation University of China, Tianjin 300300, China; jmyang@cauc.edu.cn (Z.Y.); zzc7465221@163.com (Y.D.); 2 Aeronautical Engineering Institute, Civil Aviation University of China, Tianjin 300300, China; fb062425@163.com

**Keywords:** fused deposition modeling, discrete modeling, process simulation, thermal analysis, thermo-mechanical coupling analysis

## Abstract

Fused deposition modeling (FDM), as one of the most widespread and cost-effective additive manufacturing (AM) technologies, faces ongoing challenges in improving the dimensional accuracy and mechanical properties of complex shapes. The repeated heating and cooling of thermoplastic filaments make the FDM parts prone to accumulating warping deformation, which is difficult to predict due to the specificity of material deposition toolpaths. In this study, a path-based discrete modeling and process simulation method was developed for the FDM process. Based on process parameters and material deposition toolpaths, the finite element (FE) model was reconstructed using the discrete modeling method. Then, the birth–death element method (BDEM) was employed to simulate the FDM process and solve the thermo-mechanical coupling field in ANSYS 2022 R1. The corresponding computing programs were compiled in C++. The effectiveness of the proposed method was verified by three numerical examples using ABS material. According to the results, the simulated deformations show strong agreement with the deformations of real FDM parts. The findings of this study are applicable to other mainstream AM processes and are unrestricted by any complex geometries.

## 1. Introduction

Additive manufacturing (AM) has found extensive use in the integral forming of lightweight and complex structures across multiple industries [1,2]. Compared to subtractive manufacturing, AM’s prevalence stems from its path-based material deposition process, which enables the rapid prototyping of complex geometries with high production efficiency and at a low cost [3,4]. Nevertheless, the unique feature of layer-by-layer has its own set of challenges. Structural defects, including warping, deformation, and layer delamination, are likely to occur. These issues reduce the dimensional accuracy of the final products and compromise their mechanical performance [5,6]. At present, AM remains restricted to the production of non-load-bearing structures or light industrial products [7]. To break through this bottleneck, a number of studies have delved into the structural defects of AM parts and the influencing factors. For instance, Chen et al. [8] investigated the typical structural defects occurring in the AM process, including cracking, porosity, and balling, and summarized the mainstream defect detection methods such as infrared imaging defect detection, ultrasonic defect detection, and machine learning-based defect detection technology. Xu et al. [9] proposed an improved YOLO v4 algorithm model based on a deep learning algorithm, which has higher detection accuracy and detection speed to identify the defect location of AM parts. Through statistical analysis of defect types and possible causes, the toolpath changes and parametric changes in the slicer program were utilized to train the learning model. Khosravani et al. [10] explored the effects of structural defects and accelerated thermal aging on tensile strength, stiffness, and surface roughness of AM parts. For intact and defective parts with different print orientations, it was investigated whether thermal aging would have different effects on part performance. Mohd et al. [11] used the Taguchi approach to optimize the process parameters of FDM to improve the dimensional accuracy of ABS printed parts. An orthogonal array of L’9 was designed to test four process parameters. By comparing the results, it was concluded that the optimal process parameters were honeycomb print pattern, 0 y-axis orientation, 90° support angle, and 0.4 sidewalk offset. The shrinkage of the printed part decreased from 0.619 mm to 0.429 mm.

Recent studies on AM primarily focus on using numerical simulation tools to analyze structural defects and mechanical properties. The aim is to reduce the cycle time and research cost [12,13]. Still, there exist limitations in using existing finite element (FE) analysis tools to simulate the manufacturing process based on predefined toolpaths, for example, the lack of FE models with high prediction accuracy, the study of structures that are usually simple, and the lack of universal simulation methods [14,15]. Most studies take the toolpaths as the starting point [16]. For instance, Hachimi et al. [17] converted the toolpaths programmed as G-code, a universal numerical control (NC) language for FDM, into Abaqus script to construct the real geometries. A tensile test was performed on a monolayer specimen with variable infill orientations to validate the simulation accuracy. Antonio et al. [18] developed a G-code extraction algorithm and exploited the sweep command provided by CAD to reconstruct a 3D solid structure based on a 2D profile. Andrew [19] designed an open-source software called Full-Control G-code Designer, which allows users to customize the toolpaths without relying on CAD or slicing software. Through the Excel front-end, users can easily carry out parametric design and generate G-code files, which improves the flexibility and repeatability of the design. The extensive application of Full-Control is demonstrated through several cases, including printer calibration, complex structure manufacturing, and industrial applications. Apart from AM, similar processing of G-code was studied in other manufacturing technologies. For instance, Gahbiche et al. [20] translated the toolpaths for the incremental sheet forming process from G-code into a python script, which was used as input for the Abaqus simulation. The accuracy was verified by comparing the global shape and thickness evolution with the experimental results. Zeradam and Arkanti [21,22] proposed the bidirectional translation method between the G-code and the coordinate points of forming paths for the Single Point Incremental Forming (SPIF). The extracted coordinates were utilized to numerically simulate the forming process. Zhang et al. [23] proposed a JavaScript-based method that converts images or text into G-code and optionally examines the rationality of the generated toolpaths, thereby customizing the process for precise CNC machining.

From the perspective of AM process simulation, Tawfik et al. [24] developed a 3D FE model to simulate the selective laser melting (SLM) process. It was found that the higher scanning speeds may lead to lower surface temperatures but larger vertical distortions between subsequent layers. The effects of laser scanning speed on temperature distribution and residual deformation were analyzed. Mashhood et al. [25] developed a multi-physics field model for the metal AM processes based on the FE method and G-code, which can evaluate the residual deformation and stresses. Yue et al. [26] employed Abaqus to simulate the electron beam melting (EBM) process with pure tantalum as the material. The established model can predict the temperature field distribution, molten pool evolution, and residual stress distribution, which provides a reference for adjusting process parameters in the actual AM process. Zhang et al. [27] employed the FE method to simulate the temperature field of a rectangular thin plate during the FDM process. The temperature gradient distribution at different printing speeds was analyzed. Akbar et al. [28] simulated the FDM for 4D printed shape memory polymers and optimized process parameters to reduce the built-in internal stresses and warpage by Abaqus. Similarly, Syrlybayev et al. [29] developed a thermodynamic model for the FDM process using the FE method to reduce warpage. Samy et al. [30] simulated the FDM process by COMSOL to study the influence of process parameters on residual stress and warping deformation. The results showed that reducing the delamination height and adjusting the filling method can reduce the residual stress and structural defects. Schmidt and Kyosev [31] simulated the FDM process in the python-pybullet environment by following the G-code, which discretized the molten polymer and infiltrated it into the porous substrate. The shape and penetration of the polymer were validated through experimental results. Lei et al. [32] employed PLA/GNPs nanocomposites as FDM material and studied the cross-sectional morphology and dimension of filaments under different process parameters. The influence of process parameters on the mechanical properties of FDM specimens was investigated through numerical simulation and experiments. The findings indicated that an appropriate combination of process parameters can improve the manufacturing accuracy and mechanical properties of the specimens.

To the best of the authors’ knowledge, there is still a lack of a universal process simulation method for complex geometries. Existing studies mostly concentrate on basic applications of AM simulation in simple thin plates. When process parameters undergo changes, the structure of the printed part is bound to be affected. As a result, the CAD model must be rebuilt to accurately simulate the manufacturing process. This inevitably leads to a substantial amount of repetitive work. To address this issue, this study proposed a path-based discrete modeling and process simulation method for the FDM process, which starts from the CAM stage. Firstly, the CAD model is input into the slicing software, and the process parameters can be customized according to user demands. After generating the corresponding toolpaths, the model can be automatically reconstructed through the path-based discrete modeling method. Then, the FDM process is simulated in ANSYS 2022 R1 based on the toolpaths. The thermo-mechanical coupling analysis was carried out to solve the internal stresses and warpages caused by uneven heating of the material. Therefore, it can be seen that the simulation process is entirely based on process parameters and toolpaths, which circumvents the repeated design of CAD models, realizes the rapid simulation of complex geometries under different process parameters, and meets the actual needs of personalized manufacturing. In addition to FDM, the proposed method is adaptable to other mainstream AM processes.

The rest of this paper is organized as follows. Section 2 first presents the framework of the proposed method. Then, the details of the discrete modeling method in terms of process parameters and toolpaths are described, followed by the theoretical derivation of thermo-mechanical coupling calculation for the FDM process simulation. In Section 3, three numerical examples are demonstrated to validate the reliability of the proposed method. Finally, the conclusions are given in Section 4.

## 2. Methodology

### 2.1. Framework of Path-Based Discrete Modeling and Process Simulation Method

**Figure 1:**
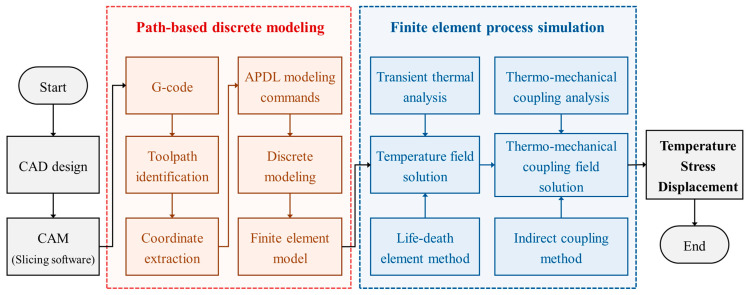
Framework of path-based discrete modeling and process simulation method.

This study proposes a path-based discrete modeling and process simulation method. The aim is to provide a universal simulation workflow for FDM applicable to complex geometries. Figure 1 depicts the framework of the proposed method, which is composed of two key parts: path-based discrete modeling and FE process simulation. The following briefly describes each part.

Path-based discrete modeling: Given that most FDM slicing software compiles machine operations and material deposition toolpaths in G-code format [33], this study used the G-code toolpaths for the discrete modeling process. During the prefabrication phase, a CAD model was imported into CAM (i.e., slicing software) and layered according to the preset process parameters. By using the built-in algorithms, the paths for controlling nozzle movement on each layer were planned and programmed into G-code. To transform all toolpaths into an FE model, the starting and ending coordinates of each toolpath were identified and extracted from the original G-code file. Subsequently, the modeling commands in ANSYS Parametric Design Language (APDL) were created. By executing each command, the FE model was reconstructed in the form of stacked material filaments.

FE process simulation: Once the reconstructed FE model was obtained, the birth–death element method (BDEM) was employed to activate the designated model element in sequence. As each element was activated, thermal loads were applied. Then, transient thermal analysis and thermo-mechanical coupling analysis were conducted in sequence via the indirect coupling method. The resulting distributions of temperature, equivalent stress, and displacement were used to predict the potential warping deformation of real FDM parts.

The corresponding C++ scripts were interpolated to translate G-code into APDL and to call the built-in mechanical solver in ANSYS to simulate the FDM process. Details of the discrete modeling method and process simulation method are presented in the following sections.

### 2.2. Path-Based Discrete Modeling Method

This section introduces the path-based discrete modeling method to reconstruct the FDM structure for FE simulation. The method consists of two steps: extracting toolpath coordinates from G-code and discrete modeling based on toolpaths.

#### 2.2.1. Extraction of Toolpath Coordinates

In the prefabrication phase of FDM, the toolpaths for depositing materials are encoded as G-code, a commonly used NC language, to command machine operations including machine heating, nozzle movement, and material extrusion [33]. Table 1 displays the typical G-code commands used to control FDM machines. To convert the material deposition toolpaths into APDL, the commands for linear nozzle movement must be filtered and processed.

**Figure 2:**
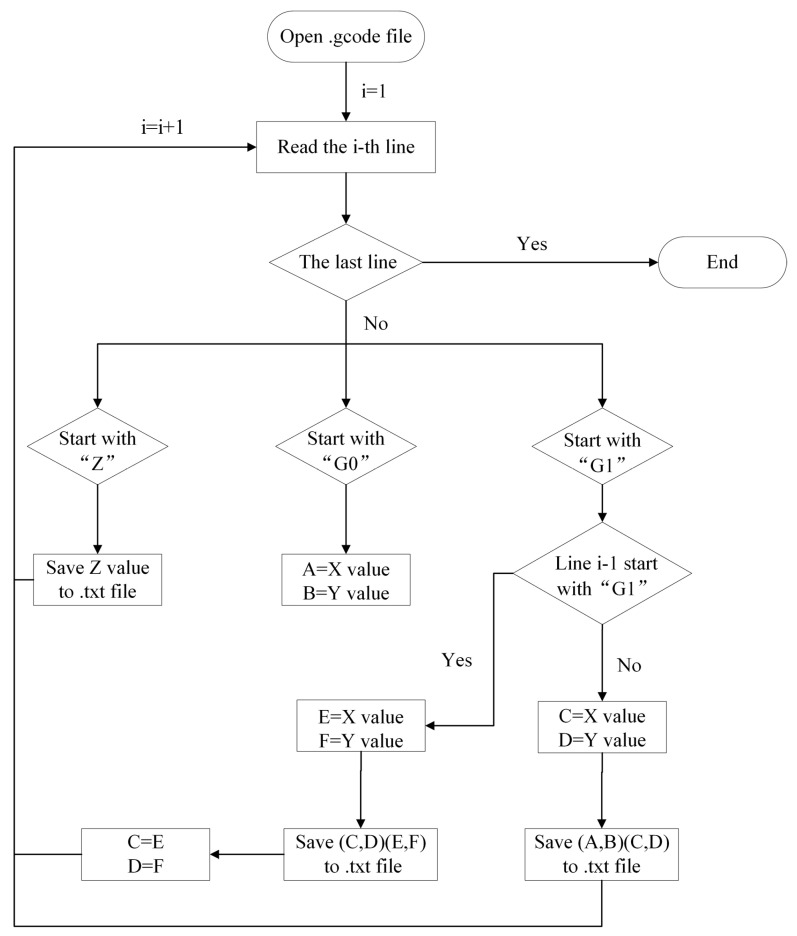
Workflow of toolpath coordinate extraction for discrete modeling.

As shown in Figure 2, the command lines are scanned one by one. When a command containing “G0”, “G1”, or “Z” is encountered, the coordinate values following “X”, “Y”, and “Z” are extracted, and only the coordinates of toolpaths with actual material extrusion are retained. Note that the command containing “Z” signifies a switch to the next layer. In this case, the target coordinate (i.e., the ending point) on the Z axis is saved to the output file. In other cases, when a command starts with “G0”, parameters A and B are set as the target coordinates on the X and Y axes, respectively. When a command starts with “G1”, two situations need to be discussed separately.

If the preceding command does not start with “G1”, assign parameters C and D as the target coordinates on the X and Y axes, respectively. Then, save the starting point (A, B) and the ending point (C, D) of the current toolpath with material extrusion to the output file.

On the contrary, if the preceding command also starts with “G1”, assign parameters E and F as the target coordinates of the current toolpath on the X and Y axes, respectively. Then, save the starting point (C, D) and the ending point (E, F) of the current toolpath to the output file. Simultaneously, update the starting coordinates of the next toolpath with the ending coordinates of the current one, i.e., C = E, D = F.

**Figure 3:**
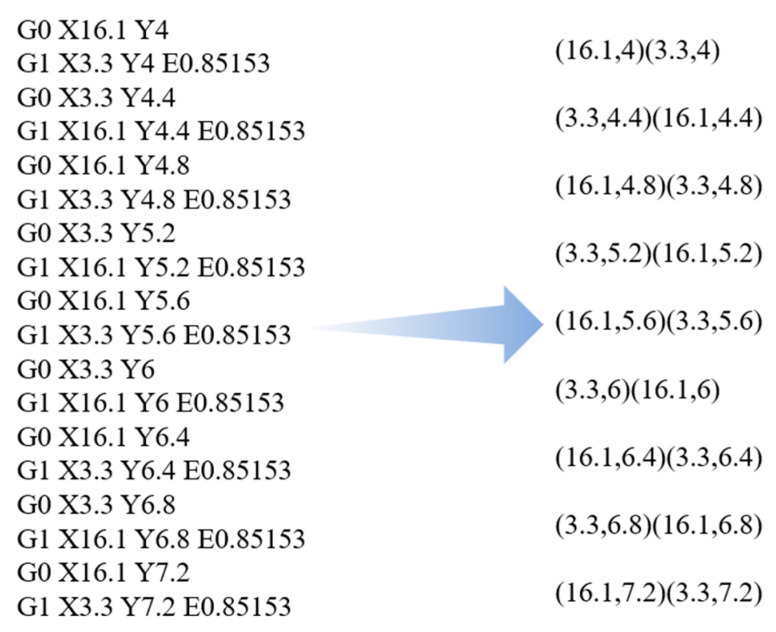
Example of toolpath coordinate extraction.

Repeat the above steps until the last command line is scanned. Finally, an output file containing the coordinate data of all material deposition toolpaths will be obtained. This file serves as input for the next step: discrete modeling. Figure 3 displays an example of the extracted coordinates. The corresponding computing program was compiled in C++.

#### 2.2.2. Path-Based Discrete Modeling

During the FDM process, material filaments are melted into a liquid state and deposited along the toolpaths through the hot end. The cross-section of the deposited filament is approximately a flat ellipse. In this study, for simplicity, the material filament was modeled as a hexahedron of a specific length to ensure contact surface between adjacent filaments, thereby guaranteeing the continuity of heat transfer during FE simulation.

Following the above simplification, the next step is converting the path coordinates into APDL-compatible instructions. The FE model is reconstructed by successively creating and accumulating elements. Note that the element size is predefined in advance and remains constant. Each element is created using the command “BLOCK, X1, X2, Y1, Y2, Z1, Z2”. Here, “X1, X2”, “Y1, Y2” and “Z1, Z2” define the geometric dimensions of the element in the X, Y, and Z directions, respectively. Z1 and Z2 denote the Z-coordinates of the bottom and top surfaces of the current layer, respectively.

**Figure 4:**
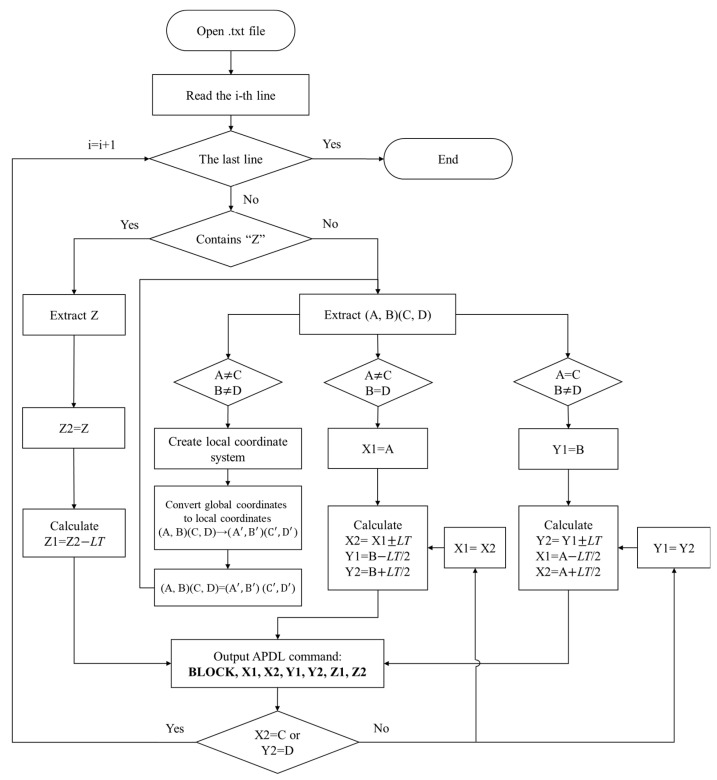
Workflow of path-based discrete modeling.

**Figure 5:**
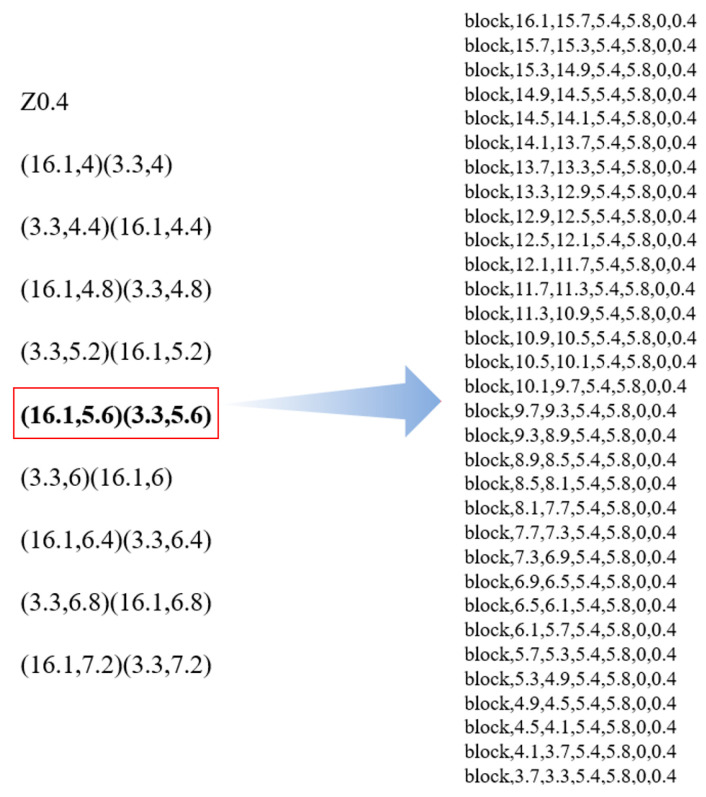
Example of converting toolpath coordinates to APDL.

To automate the conversion of all extracted coordinates into APDL modeling commands, a computing program was compiled in C++. The workflow is shown in Figure 4. First, each line of the extracted coordinates is scanned. If a line starts with “Z”, the coordinate that follows represents the top coordinate Z2 of the current layer, while the bottom coordinate Z1 is calculated as Z2 minus the layer thickness *LT*. If a line is in the format of “(A, B) (C, D)”, it indicates that the toolpath is from (A, B) to (C, D). In this case, the coordinates “X1, X2” and “Y1, Y2” of each element on the toolpath can be calculated through incremental addition or subtraction based on the element size. In this study, the length, width, and height of each element are all equal to *LT.* When encountering a toolpath with an inclination angle (i.e., A ≠ C, B ≠ D), it is necessary to convert the global coordinates of the starting and ending points into local coordinates before calculating the coordinates of each element. This ensures that the toolpath is laid along the vertical or horizontal direction in the local coordinate system, thereby simplifying the calculation process. Once the element coordinates are determined, the corresponding APDL command “BLOCK, X1, X2, Y1, Y2, Z1, Z2” is saved to the output file. Figure 5 shows an example of executing the above process, where a toolpath from (16.1, 5.6) to (3.3, 5.6) is translated into APDL modeling commands. Then, repeat the above steps until the last line is scanned. Finally, an output file with all the APDL commands is obtained to reconstruct the entire model.

**Figure 6:**
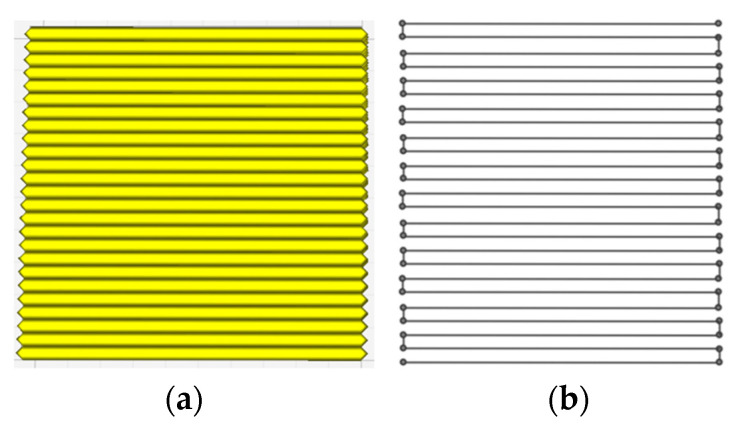
Slicing model and extracted toolpaths for discrete modeling: (**a**) slicing model; (**b**) extracted toolpaths.

**Figure 7:**
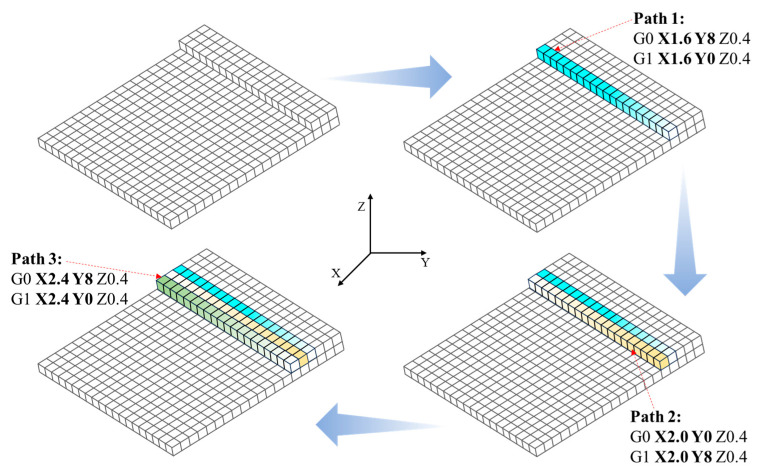
Path-based discrete modeling process.

By executing the output APDL commands, the FE model is reconstructed and ready for simulation. Figure 6 shows an example of a single-layer square plate to validate the proposed method. It was observed that the extracted toolpaths for discrete modeling are consistent with the original toolpaths in the slicing software. For the reconstructed model (see Figure 7), the modeling process is consistent with the actual FDM process, ensuring the material continuity of the entire structure.

### 2.3. Finite Element Process Simulation Method

After reconstructing the FE model, the BDEM was employed to simulate the material deposition process, followed by the simulation of the heat transfer and structural deformation. The theories and workflows of thermal analysis and thermo-mechanical coupling analysis are described in this section.

#### 2.3.1. Birth–Death Element Method

**Figure 8:**
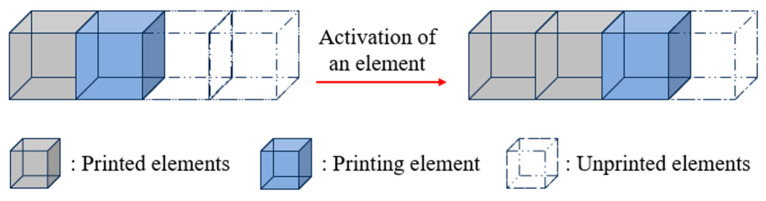
Birth and death process of structural elements.

The simulation of material deposition is achieved through the manipulation of element birth and death. To begin, all elements of the FE model are set to the “death” state. As printing begins, the specified elements are activated from “death” to “birth” by following the material deposition toolpaths, as shown in Figure 8. In an ideal scenario, non-activated or unprinted elements are assumed to be “killed” without material properties, and thus, can be considered as not participating in any heat convection or heat conduction. In the actual simulation process, it should be noted that those elements have not been truly “killed” but still exist in the FE model. To ensure that the structure has a unique deformation solution under thermal load, the stiffness matrix of any non-activated element must not be a zero matrix. Therefore, the BDEM multiplies the original stiffness matrix by a reduction coefficient to minimize the impact of non-activated elements on the simulation result. Once an element is activated, its properties, e.g., mass, stiffness, specific heat, and strain, are restored to their original values. In this study, the BDEM was used in conjunction with thermal analysis and thermo-mechanical coupling analysis. The principles and workflows are described in detail in the following sub-sections.

#### 2.3.2. Theory of Thermal Analysis

Due to the movement of the high-temperature nozzle, the FDM part undergoes repeated heating and cooling during printing. The temperature, heat flux, thermal boundary conditions, and internal energy of the printed part all vary over time. Therefore, the numerical simulation of the FDM process is a transient thermal analysis problem. According to the law of energy conservation, the transient thermal equilibrium equation is expressed In matrix form as follows:

(1) $$\left[C\right]\left\{\dot{T}\right\}+\left[K\right]\left\{T\right\}=\left\{Q\right\}$$

where $\left[C\right]$ is the specific heat capacity matrix; $\left[K\right]$ is the conduction matrix composed of convection, thermal, radiation, and shape coefficients; $\left\{T\right\}$ is the nodal temperature vector; $\left\{\dot{T}\right\}$ is the derivative of temperature with respect to time; and $\left\{Q\right\}$ is the nodal heat flow rate [34].

**Figure 9:**
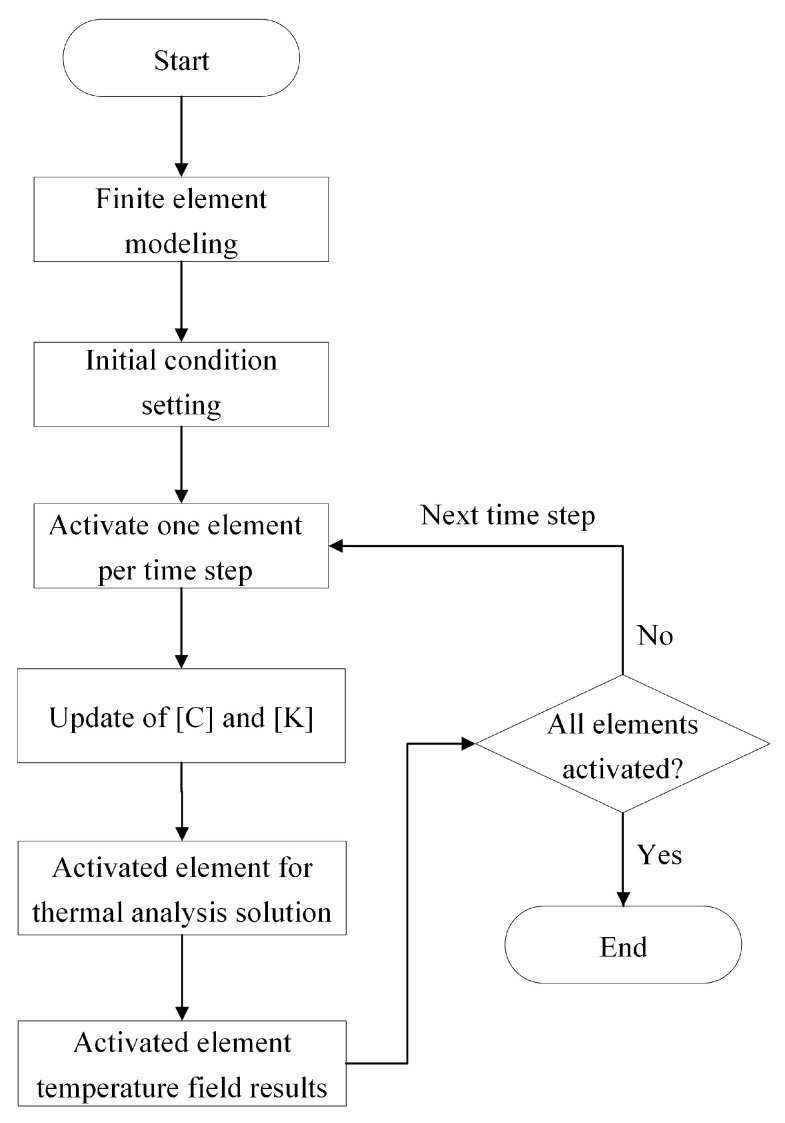
Principle of BDEM for solving the transient heat transfer problem.

To simulate the heat transfer process, it is necessary to combine Equation (1) with the BDEM. Figure 9 illustrates the basic principle of applying the BDEM to thermal analysis. First, all elements are initially “killed”, and the corresponding stiffness matrices are multiplied by a reduction factor, thus being approximated as not participating in heat transfer calculations. As each element is activated in time steps, the stiffness matrix is restored to the original state, and the matrices $\left[C\right]$ and $\left[K\right]$ for the entire FE model are updated. Then, the nodal temperatures are solved using the updated $\left[C\right]$ and $\left[K\right]$ through Equation (1). Simultaneously, the temperature field in the current time step is obtained by using the implicit time integration method. The iteration ends when the last element is activated.

In addition, the initial conditions, i.e., the temperature distribution at the beginning of printing, need to be defined in advance:

(2) $$T{|}_{t=0}=T_{0}(x,y,z)$$

**Figure 10:**
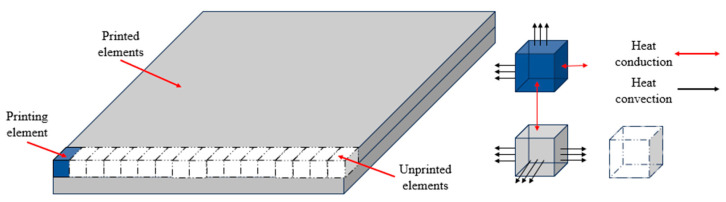
Heat conduction and heat convection in the FDM process.

In the FDM process, there are two forms of heat exchange: heat conduction and heat convection. The former refers to the heat transfer process between the printed material and the molten material to be extruded. The latter refers to the heat transfer process between the printed material and the surrounding air medium. As shown in Figure 10, heat conduction occurs at the interface between adjacent material elements, while heat convection occurs on the contact surface between the printed elements and the air. Therefore, the boundary conditions for these two types of heat exchange need to be defined:

Heat conduction: the temperature distribution at the boundary of the printed part:
  (3) $$T{|}_{s}=T_{s}(x,y,z,t)$$
  where $T_{s}(x,y,z,t)$ denotes the time-varying temperature of activated elements that have been previously solved. The subscript S  denotes the boundary of the printed part.
Heat convection: the convective heat transfer between the printed part and air medium in contact:
  (4) $$k_{x}\frac{\partial T}{\partial x}n_{x}+k_{y}\frac{\partial T}{\partial y}n_{y}+k_{z}\frac{\partial T}{\partial z}n_{z}=h\left(T_{\alpha }-T\right)\;$$
  where $k_{x}$, $k_{y}$, $k_{z}$ denote the thermal conductivity in X, Y, Z directions, respectively; $n_{x}$, $n_{y}$, $n_{z}$ denote the directional cosines of the boundary normal; *h* denotes the convective heat transfer coefficient; and *T* denotes the surface temperature of the printed part. The meaning of $T_{\alpha }$ depends on the thermal convection conditions. Under natural convection conditions, $T_{\alpha }$ denotes the ambient or medium temperature; under forced convection conditions, $T_{\alpha }$ denotes the wall temperature of the boundary layer.

**Figure 11:**
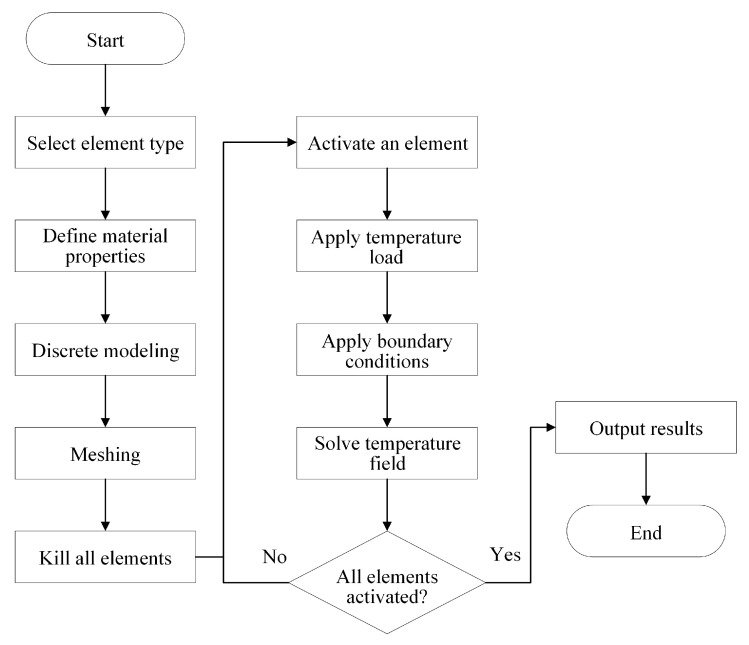
Workflow of FDM transient thermal analysis.

Based on the above theories, the complete workflow for solving the FDM temperature field is presented in Figure 11. To begin, the toolpaths for printing the CAD design are planned through the slicing software. Using the predefined element type and material properties, the corresponding FE model is reconstructed via the proposed path-based discrete modeling method. The mesh of the FE model can be further refined according to the simulation accuracy requirements. At the beginning of the simulation, all elements are initially “killed” and then activated one by one following the toolpath trajectories. When an element is activated, a thermal load representing the high-temperature nozzle is applied to the element, followed by the boundary conditions for heat conduction and convection. When the next element is activated, the thermal load is moved to that element. The calculation results from the previous step serve as the initial condition for solving the transient temperature field in the current step. Once all the elements are activated, the temperature field simulation results of the entire FDM part can be obtained.

#### 2.3.3. Theory of Thermo-Mechanical Coupling Analysis

The actual FDM process features a large number of toolpaths. As a result, the corresponding finite element (FE) model contains an enormous number of element nodes. For an FE model with a high degree of freedom, the indirect coupling method is well suited for addressing the coupling issues among multiple physical fields. In this study, this method was based on the temperature field solved in the previous section. Specifically, the temperature field results were applied as external loads to the mechanical field for further solutions. To start with, the following assumptions have been made:

Material continuity: Assuming that the material filaments do not break or overlap during deformation. The deformation and internal force of the FDM part are continuous functions of coordinates.

Homogeneity and isotropy: assuming that the material filaments are homogeneous, and the created material elements have the same mechanical properties in any direction.

Small deformation: assuming that the material deformation obeys the small strain criterion. The stress–strain relationship is linear within a short period.

Von Mises stress yield criterion: assuming that plastic deformation occurs when the internal stress of the material reaches its limit value.

To solve the thermo-mechanical coupling problem in this study, the equivalent stress yield criterion was used to characterize the material under plastic deformation. The mathematical expression is given in the following equation:

(5) $$\bar{\sigma }=\frac{\sqrt{2}}{2}\sqrt{(\sigma_{1}-\sigma_{2}{)}^{2}+(\sigma_{2}-\sigma_{3}{)}^{2}+(\sigma_{3}-\sigma_{1}{)}^{2}}$$

where $\sigma_{1}$, $\sigma_{2}$, $\sigma_{3}$ are the first, second, and third principal stresses, respectively.

During the FDM process, as the temperature changes, the material filament transitions from the glassy state to the molten state and then reverts to the glassy state. The variation in material physical properties leads to the generation of small strain increments $d\left\{\varepsilon \right\}$, including three strain components:

(6) $$d\left\{\varepsilon \right\}=d\left\{\varepsilon_{e}\right\}+d\left\{\varepsilon_{p}\right\}+d\left\{\varepsilon_{T}\right\}$$

where $d\left\{\varepsilon_{e}\right\}$ is the elastic strain increment; $d\left\{\varepsilon_{P}\right\}$ is the plastic strain increment; and $d\left\{\varepsilon_{T}\right\}$ is the thermal strain increment of the material. Each component is described as follows.

Elastic strain increment: According to Hooke’s law, the relationship between elastic strain increment and stress increment is expressed as follows:

(7) $$d\left\{\sigma \right\}=\left[D_{e}\right]d\left\{\varepsilon_{e}\right\}$$

where $d\left\{\sigma \right\}$ represents the stress increment and $\left[D_{e}\right]$ is the matrix of coefficients related to Poisson’s ratio and elastic modulus. Since the elastic strain is determined by the temperature *T*, Equation (7) can be rewritten as follows:

(8) $$d\left\{\varepsilon_{e}\right\}={\left[D_{e}\right]}^{-1}d\left\{\sigma \right\}+\frac{{\left[D_{e}\right]}^{-1}}{\partial T}\left\{\sigma \right\}dT$$

Plastic strain increment: Plastic deformation occurs when the material yield reaches the limit. According to the flow theory of plasticity, the plastic strain increment is expressed as follows:

(9) $$d\left\{\varepsilon_{p}\right\}=\lambda \frac{\partial f}{\partial \left\{\sigma \right\}}$$

where f is the yield function that characterizes the relationship between stress components. λ is a scalar multiplier, namely, the plastic coefficient or loading coefficient, which is determined by the plastic deformation properties of the material.

Thermal strain increment: Thermal strain is caused by thermal expansion and contraction. The reason is that variations in material temperature affect the energy-driving molecular thermal motion, thereby altering the intermolecular interaction forces and causing structural deformation. The thermal strain increment satisfies

(10) $$d\left\{\varepsilon_{T}\right\}=\left\{\alpha_{0}dT+Td\alpha_{0}\right\}=\left\{\alpha_{0}+\frac{\partial \alpha_{0}}{\partial T}\right\}dT=\left\{\alpha \right\}dT$$

where α is the coefficient of thermal expansion; $\alpha_{0}$ is the coefficient of thermal expansion at the initial temperature; and T is the instantaneous temperature [35,36].

**Figure 12:**
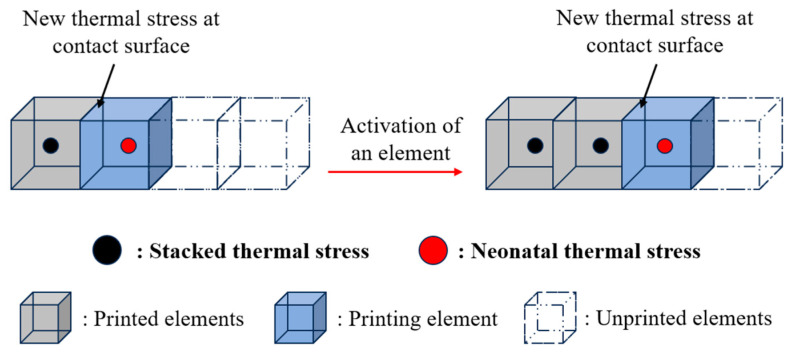
Generation of thermal stress during the FDM process.

The deformation mechanism of the FDM structure can be deduced from the above equations. As molten material is deposited, heat conduction occurs between adjacent elements. The newly deposited material experiences a significant temperature drop, leading to more substantial shrinkage compared to adjacent materials. As a result, thermal stress is generated at the contact surface, as shown in Figure 12. In addition, as the high-temperature nozzle moves continuously over the printed part, the entire structure undergoes repeated heating and cooling cycles, which results in the accumulation of internal thermal stress and intensifies the generation of deformation.

**Figure 13:**
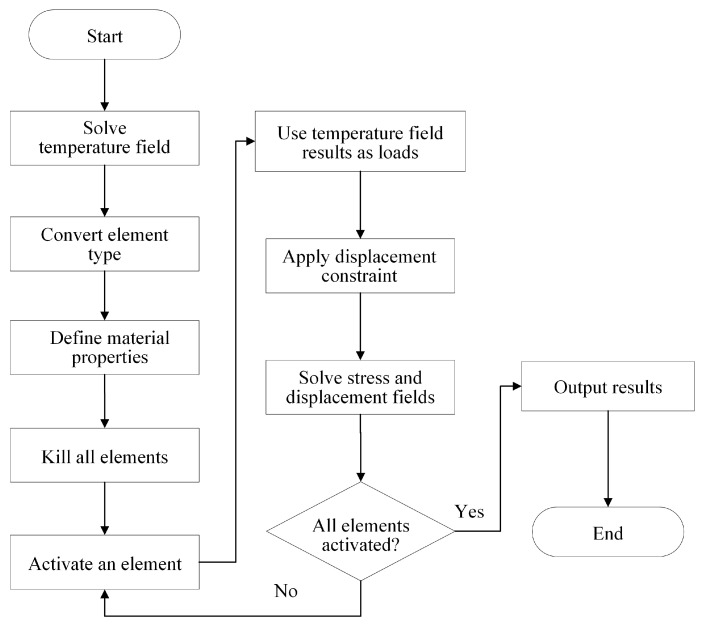
Workflow of indirect thermo-mechanical coupling method.

The workflow for solving the thermo-mechanical field is shown in Figure 13. After obtaining the temperature field results, the next step is to convert the element type of thermal analysis to that of structural analysis in order to facilitate the coupling field calculations in the APDL mechanical solver. Then, the mechanical-related material properties are defined. By applying BDEM, the initially “killed” elements are activated one by one following the toolpaths. When an element is activated, the temperature variation of this element is applied as a load, and the displacement constraint is specified. In the actual FDM process, a thin layer of adhesive is coated on the hot bed to ensure that the printed part adheres firmly to the bed. Thus, in this study, the degrees of freedom of all element nodes on the bottom surface are constrained in the X, Y, and Z directions. Then, the indirect coupling method is employed to solve the stress field and displacement field by the mechanical solver. When the next element is activated, the results from the previous step serve as the initial condition to continue the iteration. Once all the elements are activated, the simulated thermo-mechanical coupling field of the whole FDM part is obtained.

## 3. Results and Discussions

### 3.1. Finite Element Simulation and Experimental Setup

To verify the effectiveness of the proposed method, a square plate and a grid plate were designed as numerical examples. The software and hardware environment were set up to support the FE simulation, as listed in Table 2. The material used in both simulation and experiment is Acrylonitrile-butadiene-styrene (ABS) thermoplastic filament from Bambu. The thermal and mechanical properties of ABS are listed in Table 3. In the prefabrication stage, the values of major process parameters for slicing the CAD model and planning the material deposition toolpaths are shown in Table 4. Other process parameters are default.

With respect to the FE simulation, the 3D Solid70 element was selected to mesh the reconstructed model for thermal analysis. The reason is that Solid70 can create a contact surface between adjacent elements to ensure the continuity of heat transfer. For the thermo-mechanical coupling analysis, the element needs to be further converted to Solid185 to facilitate the stress and displacement calculations in the mechanical solver.

### 3.2. Application Indication

**Figure 14:**
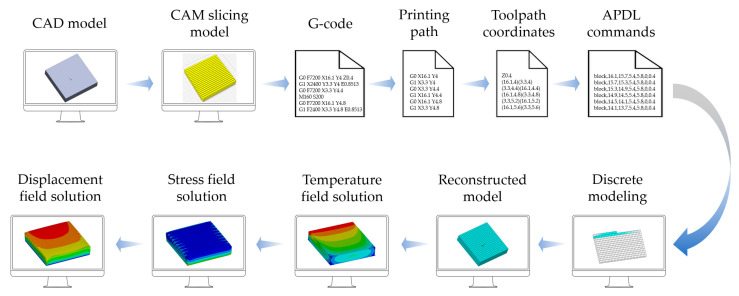
Application indication of path-based discrete modeling and process simulation.

Figure 14 demonstrates the indication of applying the proposed method to an actual FDM device. First, a CAD model is designed and saved in the STL format. The STL file is subsequently imported into the slicing software, where process parameters are configured and toolpaths are planned. Then, a G-code file is generated and processed via the path-based discrete modeling method proposed in Section 3. G-code commands related to the toolpaths are retrieved. The corresponding starting and ending coordinates are extracted. Based on these coordinates, an APDL modeling command stream is generated, which serves as the input for reconstructing the FE model. By inputting the APDL commands into ANSYS, a discrete model is reconstructed. Following the process simulation method in Section 4, the material parameters and loads are predefined, and the temperature field of the FDM process is simulated. Based on the obtained temperature field, the thermo-mechanical field is simulated to calculate the stress and displacement distributions.

### 3.3. Numercial Examples

#### 3.3.1. Square Plate Structure

**Figure 15:**
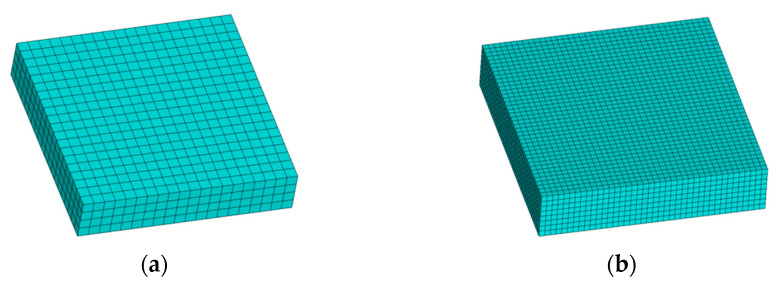
Reconstructed model and meshing model of the square plate: (**a**) reconstructed model; (**b**) meshing model.

In the first numerical example, an 8 mm×8 mm×1.6 mm square plate with four layers was designed to validate the proposed method. By applying the path-based discrete modeling method, the model was reconstructed based on material deposition toolpaths, as shown in Figure 15a. For FE simulation, the reconstructed model was further meshed with the element size of 0.2 mm×0.2 mm×0.2 mm, as shown in Figure 15b.

**Figure 16:**
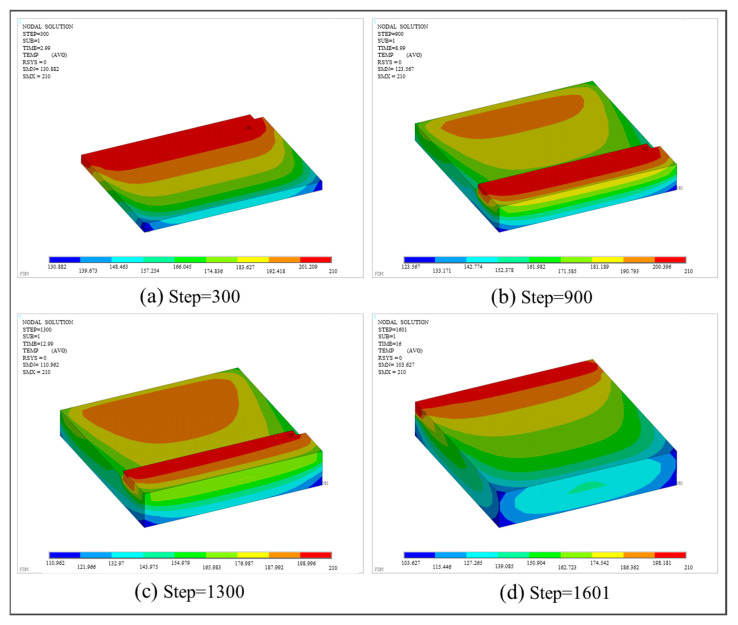
Temperature nephogram of the square plate at different load steps.

By applying the FE process simulation method, the results of thermal analysis and thermo-mechanical coupling analysis were obtained. With respect to thermal analysis, Figure 16 presents the temperature distributions at different load steps. SMX represents the highest temperature, and SMN represents the lowest temperature. It is observed that the simulated material deposition process strictly follows the predefined toolpaths. The maximum temperature of 210 °C is distributed on the newly activated elements and decreasingly dispersed to the surrounding area. Due to the bottom layer being printed first and the hot bed providing a stable heat source of 50 °C, the bottom material in contact with air dissipates heat the fastest, resulting in the lowest temperature distribution.

**Figure 17:**
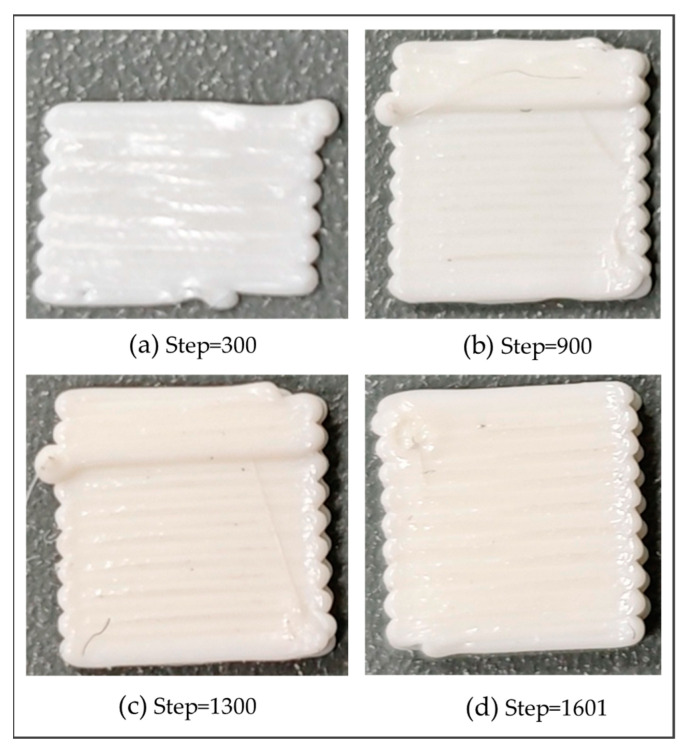
Real printing process of the square plate at different load steps.

During the simulation process, the activation sequence of elements aligns precisely with the predefined toolpaths encoded in the G-code. This sequence accurately mirrors the order of material extrusion in the actual printing process, as shown in Figure 17.

**Figure 18:**
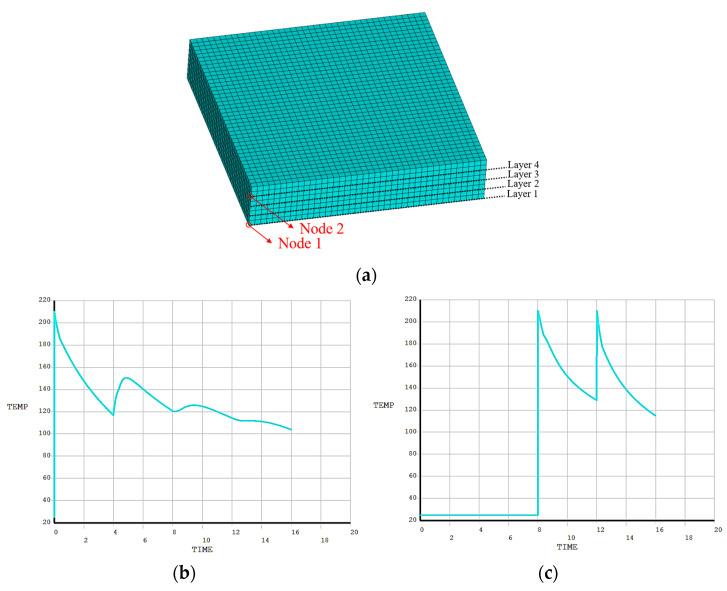
Temperature curves of random nodes on the square plate: (**a**) meshing model for FE simulation; (**b**) temperature curve of Node 1; (**c**) temperature curve of Node 2.

In addition, two nodes were randomly selected to compare the temperature variations, as shown in Figure 18a. From Figure 18b, it can be observed that the temperature curve of Node 1 exhibits multiple stationary values with a declining trend. The first peak temperature of 210 °C was reached when the element was activated, i.e., when the material underwent melting and deposition. As the nozzle was distanced from Node 1, the temperature demonstrated a steady downward trend as a result of heat conduction and convection. The cause of the second temperature rise is that the nozzle melted and deposited a new layer of material above the current element. The thermal conduction between adjacent materials led to a temperature rise at Node 1. Subsequently, as the nozzle moved away again, the temperature gradually dropped. The greater the distance between the nozzle and Node 1, the less pronounced the temperature fluctuations became.

From Figure 18c, the temperature curve of Node 2 presents two stationary values, both reaching 210 °C. Note that Node 2 is located between the second and third layers. During the printing of the first layer, the elements of other layers were not activated, thus the temperature of Node 2 remained at room temperature. Until the beginning of the second layer printing, the nozzle passed through Node 2 and deposited material, causing the temperature to reach its first peak. When printing the third layer, the nozzle passed through Node 2 again, resulting in the second temperature peak. Based on the above, it can be concluded that the heating conditions vary across different areas of the FDM structure, and these variations are primarily determined by the toolpaths.

**Figure 19:**
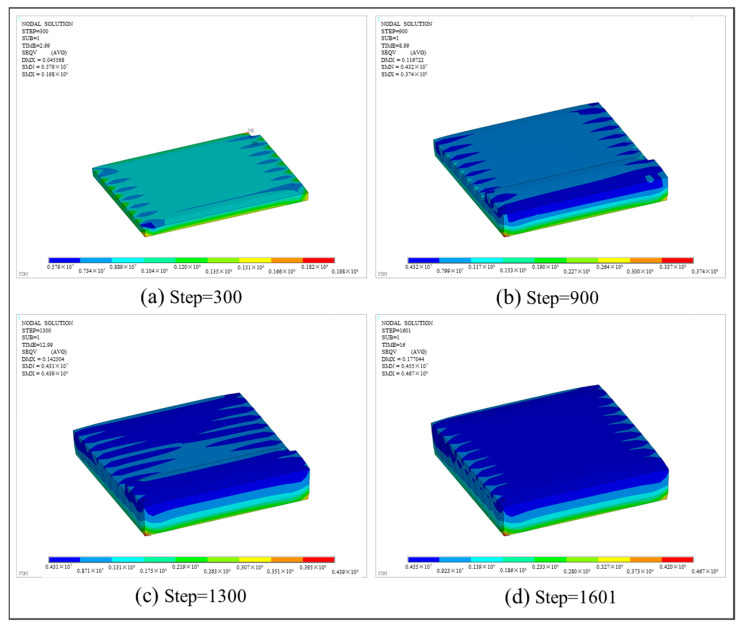
Stress nephogram of the square plate at different load steps.

With respect to thermo-mechanical coupling analysis, Figure 19 depicts the equivalent stress distributions at various load steps. SMX represents the maximum stress, and SMN represents the minimum stress. It can be observed that the maximum equivalent stress is predominantly distributed along the four sides of the bottom. The underlying reason is that the bottom layer was printed first and began to cool down. Referring to the temperature field in Figure 18, the bottom surface was in direct contact with the hot bed, while the four sides of the bottom surface were exposed to air. Thus, the material on the four sides cooled down the fastest. Given the thermal expansion coefficients of ABS at different temperatures, the free expansion and contraction of each printed layer were limited as the temperature dropped, resulting in the generation of thermal stress. Therefore, the equivalent stress at the edge of the square plate is generally higher than that in the interior area.

**Figure 20:**
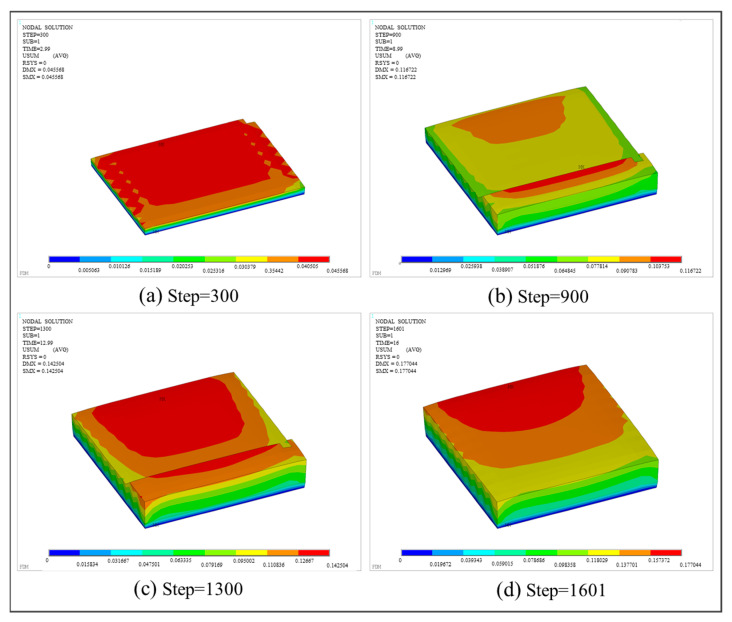
Displacement nephogram of the square plate at different load steps.

Furthermore, Figure 20 presents the displacement distributions at different load steps. DMX represents the maximum deformation of the entire structure, and SMX represents the maximum deformation at the current step. It can be observed that the structural deformation displays a gradient distribution. Due to the displacement constraint applied to the bottom surface in the X, Y, and Z directions, there was no deformation in the first layer adjacent to the hot bed. As the number of layers increased, the interlayer stress and deformation accumulated layer by layer, resulting in the maximum deformation concentrated in the last printed area and the upper edge of the square plate.

**Figure 21:**
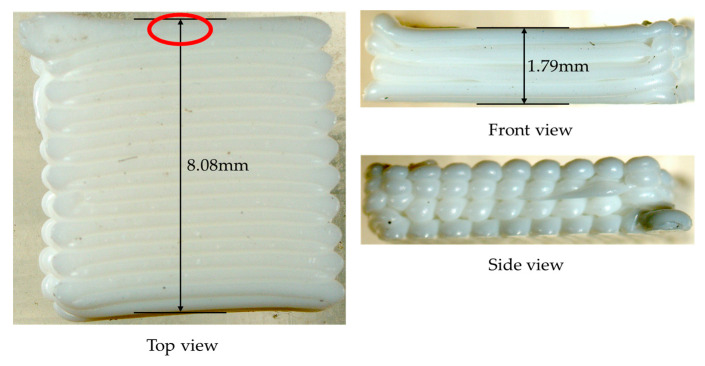
Three views of the real printed square plate.

To verify the correctness of the simulation results, the square plate was printed using the Bambu Lab X1-Carbon model 3D printer, with the same toolpaths and process parameters as the FE simulation. To clearly observe the structural deformation, the real printed structure was photographed with an optical microscope, as shown in Figure 21. It can be seen from the front and side views that obvious warping deformations accumulate at both ends of each toolpath and there is delamination between adjacent layers. The overall structure presents a shape where the middle part is equally thick and smooth, while the edges are warped upwards. Those are due to the uneven material extrusion from the nozzle and the accumulated thermal stress caused by the uneven heating of the structure.

In the numerical example, the geometry of the material filament was simplified as a hexahedron. In actual printing processes, however, the cross-section of the material filament is elliptical. Therefore, the model exhibits minor deformation within the X-Y plane, as shown from the top view and the side view in Figure 21. With respect to the front view, the left side of the model exhibits more significant deformation. This is due to the relatively small size of the model. Each toolpath initiated from the right side of the model and terminated on the left side. At the end of each path, the nozzle ceased material supply. As a result, material accumulated on the left side of the model, leading to excessive deformation of this part.

As shown in Figure 20d, the maximum deformation of 0.177044 mm occurs at the mid-section of the model upon completion of printing. To validate the consistency between this simulated deformation and the actual deformation, a vernier caliper was employed to measure the deformation of the printed part. At the same position, as marked by the red circle in Figure 21, the measured total thickness of the printed part is 1.79 mm, whereas the thickness of the CAD model is 1.6 mm. Thus, the printing-induced deformation amounts to 0.19 mm, with a relative error of 7.32% compared to the theoretical value, which proves the feasibility and reliability of the proposed method.

#### 3.3.2. Grid Plate Structure

**Figure 22:**
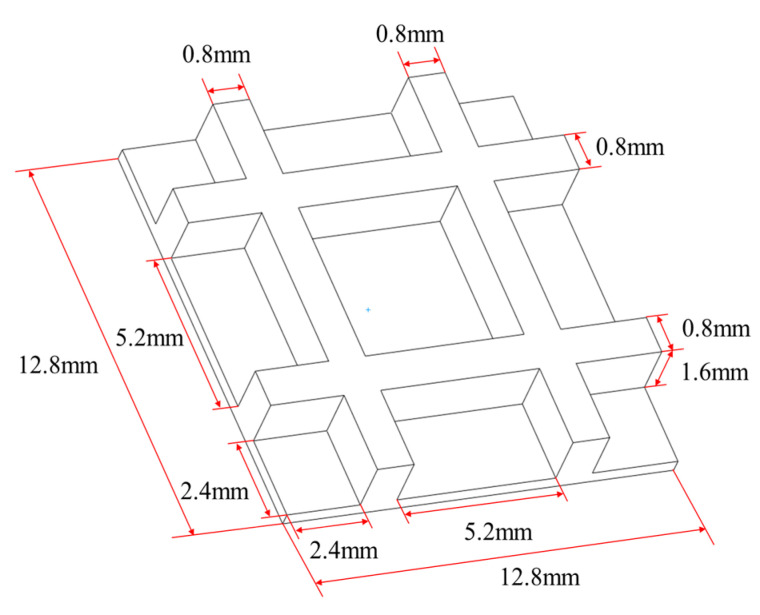
Dimensions of the grid plate.

**Figure 23:**
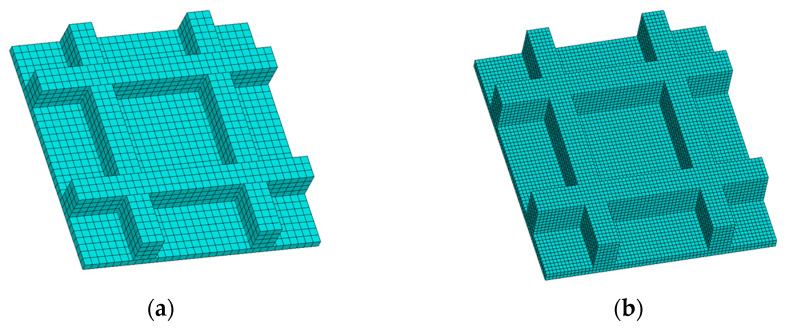
Reconstructed model and meshing model of the grid plate: (**a**) reconstructed model; (**b**) meshing model.

In the second numerical example, a grid plate with five layers was designed to validate the proposed method, as shown in Figure 22. Through the proposed method, the model was reconstructed and further meshed with the element size of 0.2 mm×0.2 mm×0.2 mm, as shown in Figure 23.

**Figure 24:**
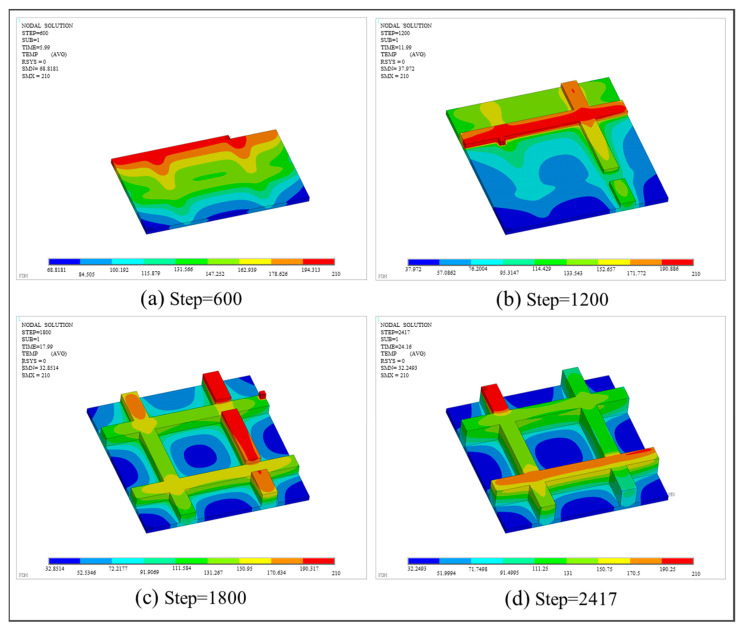
Temperature nephogram of the grid plate at different load steps.

With respect to thermal analysis, the temperature fields at various load steps are shown in Figure 24. Similar to the first numerical example, the toolpaths of FE simulation are in congruence with the predefined toolpaths. The lowest temperatures are concentrated on the first printed base plate. Conversely, the maximum temperatures are distributed on the latest printed grid and dissipated in a decreasing manner to the surrounding regions. In Figure 24a,b, note that heat dissipation is slow at the intersection of the base and the grid when grid printing has not yet commenced. The reason is that the non-activated elements were not truly “killed”. Instead, as elaborated in Section 2.3.1, the corresponding stiffness matrices were multiplied by a reduction coefficient to reduce their impact on the transient thermal calculation. Such an impact can only be weakened but is unable to be eliminated. As a result, when activated elements share nodes with non-activated elements, the heat dissipation of the activated elements is relatively slowed down. This phenomenon is common in FE simulations that employ the BDEM. Given that most AM simulation studies mainly focus on simple symmetrical structures, the slow heat dissipation is not pronounced. Its impact on the simulation result is weak and, consequently, often neglected.

**Figure 25:**
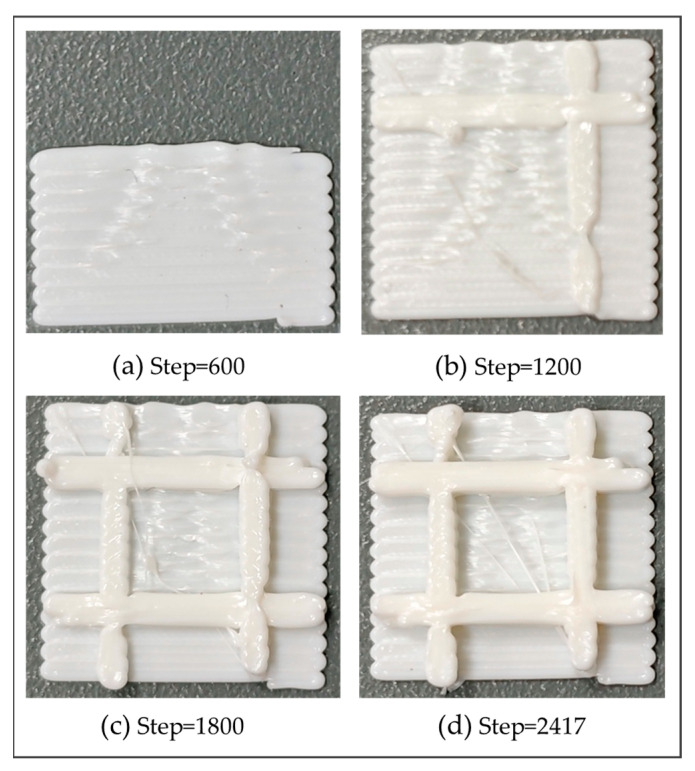
Real printing process of the grid plate at different load steps.

**Figure 26:**
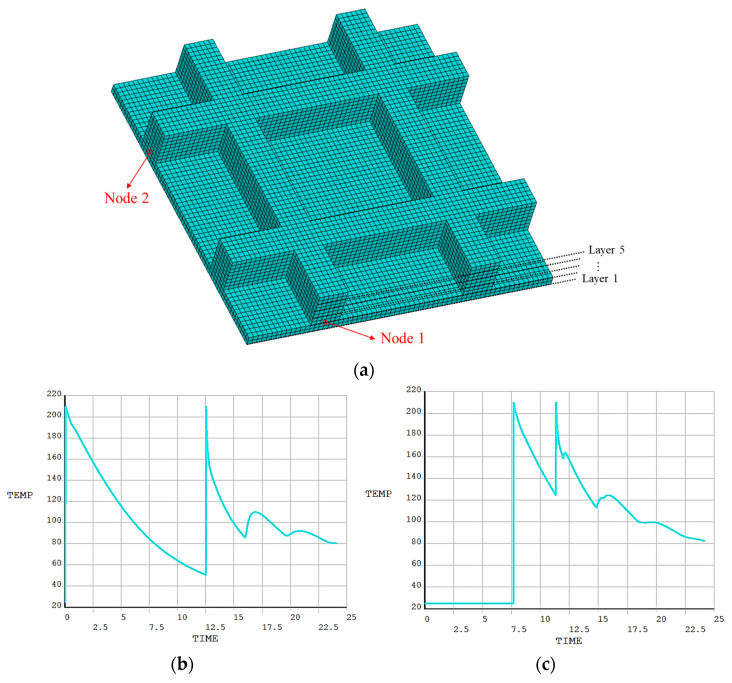
Temperature curves of random nodes on the grid plate: (**a**) meshing model for FE simulation; (**b**) temperature curve of Node 1; (**c**) temperature curve of Node 2.

Similar to the first numerical example, it was found that the simulated FDM process is completely identical to the actual printing process, as shown in Figure 25. Two nodes were randomly selected to compare the temperature variation, as shown in Figure 26a. From Figure 26b,c, it is observed that the temperature curves of both nodes have two evident peaks, which were caused by the high-temperature nozzle depositing materials next to the nodes. 

**Figure 27:**
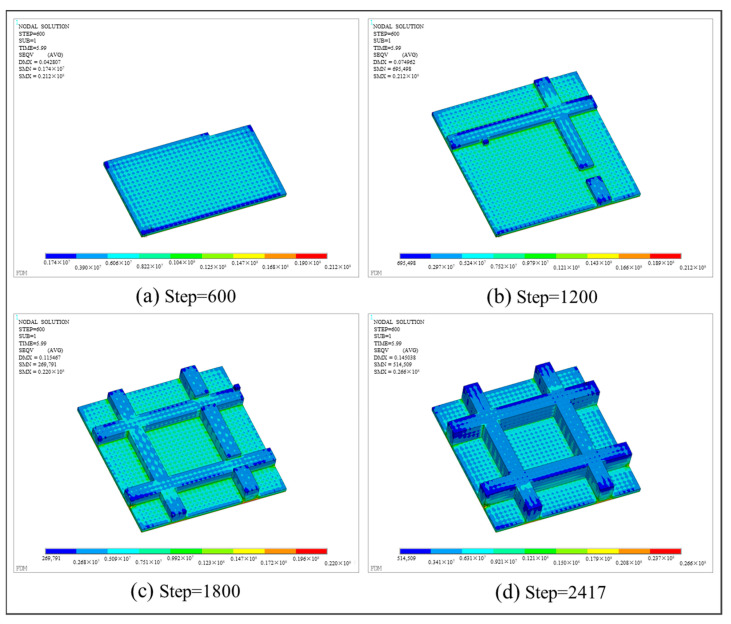
Stress nephogram of the grid plate at different load steps.

**Figure 28:**
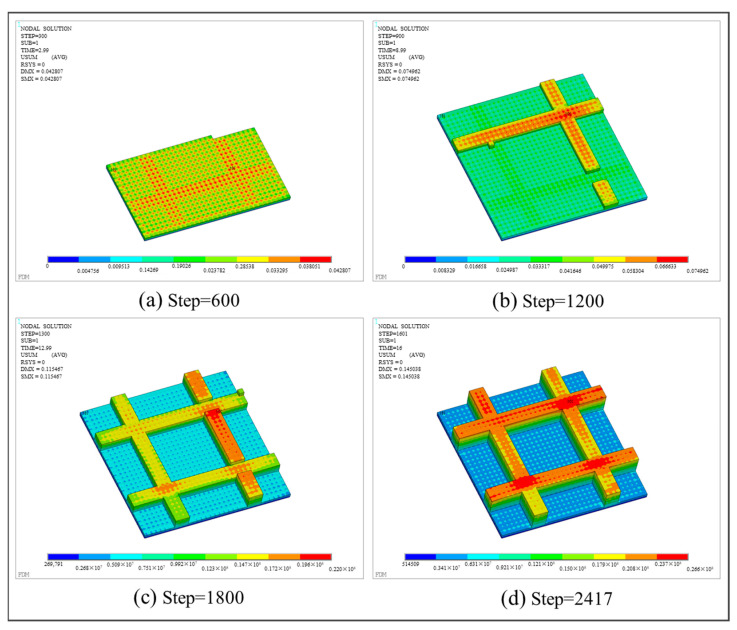
Displacement nephogram of the grid plate at different load steps.

With respect to thermo-mechanical coupling analysis, the distributions of equivalent stress and displacement are shown in Figure 27 and Figure 28, respectively. Different from the square plate, the maximum equivalent stress of the grid plate is mainly distributed at the junction of the grid and the base. This is because the amount of material needed for each layer of the grid is significantly less than that of the base. Thus, the grid material experienced fewer repeated heating cycles and cooled more rapidly. Due to the heat exchange between the edge and the air, the first printed layer at the intersection of the grid and the base dissipated heat most quickly, resulting in the concentration of thermal stress. In addition, the displacement of the final workpiece exhibits a gradient distribution due to the layer-by-layer accumulation of deformation. As a result, the maximum deformation is concentrated on the last printed layer and along the edges of the grid.

**Figure 29:**
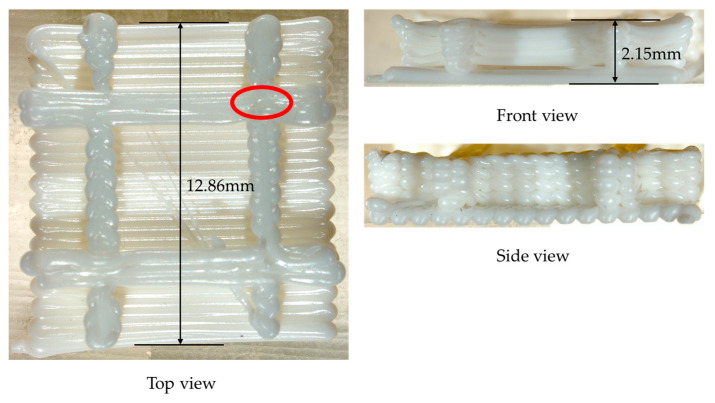
Three views of the real printed grid plate.

Compared to the real printed structure, it can be seen from Figure 29 that there is significant delamination at the junction of the grid and the base, which is due to thermal stress concentration. The warping deformation at the edges of the grid shows a layer-by-layer accumulation trend. Moreover, the model experiences small deformations within the X-Y plane, as can be seen from the top view and the side view. The maximum deformation of 0.145038 mm takes place at the junction of the grid at the end of the printing, as marked by the red circle in Figure 29. The thickness at this position measured by the vernier caliper is 2.15 mm, and the thickness of the CAD model is 2.0 mm. Thus, the actual deformation of the printed part is 0.15 mm, and the relative error relative to the theoretical value is 3.42%. It proves the consistency between the simulation result and the real printed result.

#### 3.3.3. Honeycomb Structure

**Figure 30:**
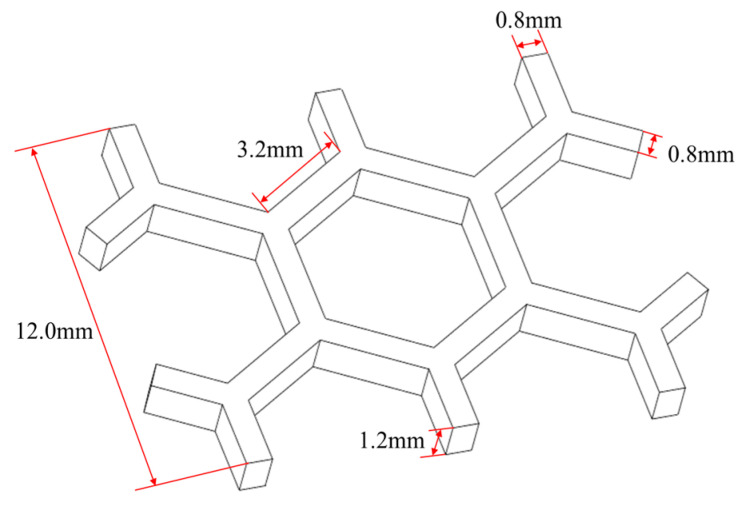
Dimensions of the honeycomb structure.

**Figure 31:**
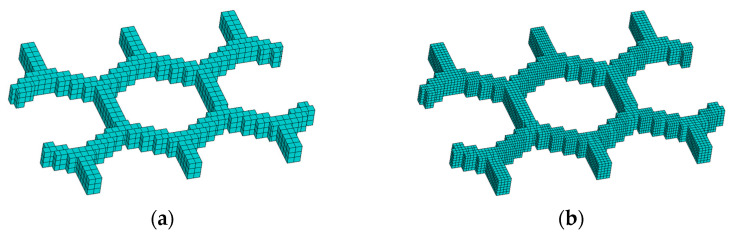
Reconstructed model and meshing model of the honeycomb structure: (**a**) reconstructed model; (**b**) meshing model.

In the third numerical example, a honeycomb structure was designed to validate the proposed method, as shown in Figure 30. Through the proposed method, the model was reconstructed and further meshed with the element size of 0.2 mm×0.2 mm×0.2 mm, as shown in Figure 31.

**Figure 32:**
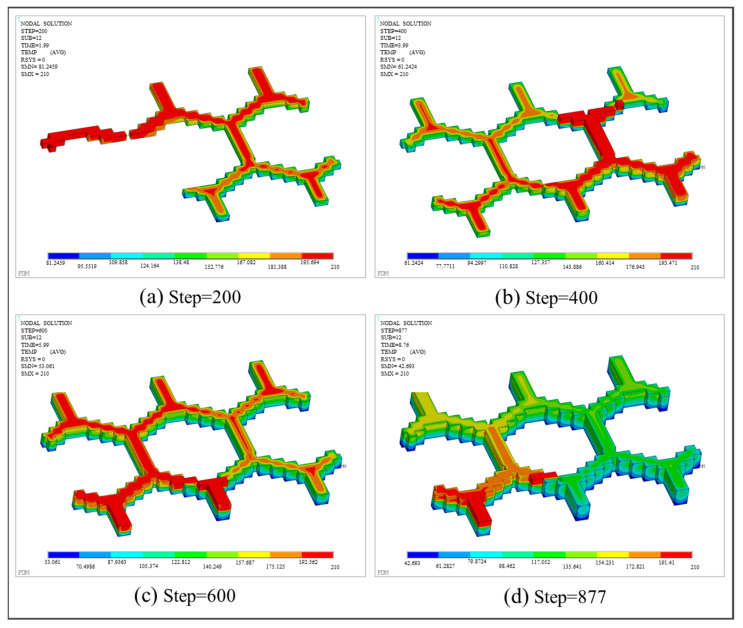
Temperature nephogram of the honeycomb structure at different load steps.

**Figure 33:**
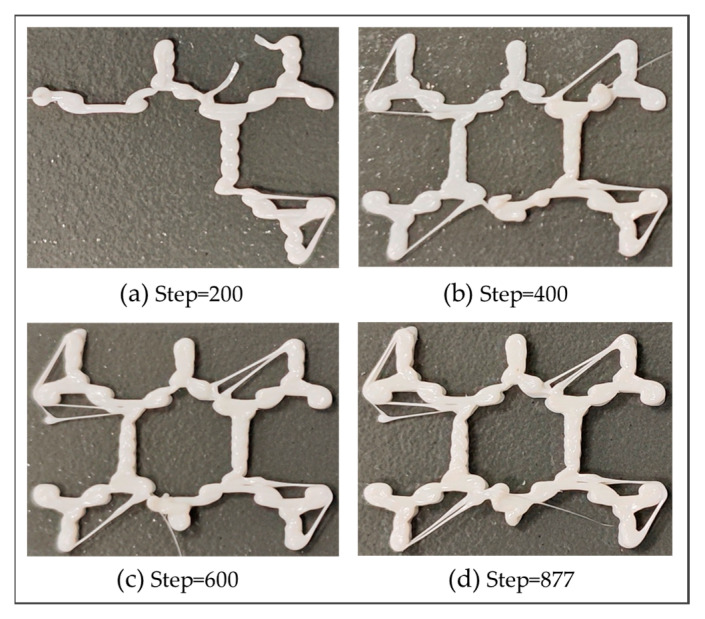
Real printing process of the honeycomb structure at different load steps.

**Figure 34:**
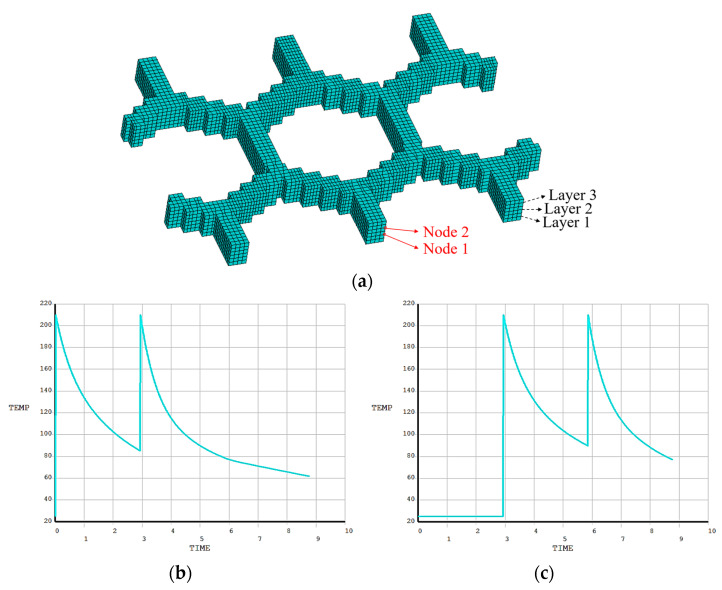
Temperature curves of random nodes on the honeycomb structure: (**a**) meshing model for FE simulation; (**b**) temperature curve of Node 1; (**c**) temperature curve of Node 2.

With respect to thermal analysis, the temperature fields at various load steps are shown in Figure 32. Similar to the previous two numerical examples, the simulation process is comparable to the actual printing process, as shown in Figure 33, which proves that the simulated FDM process is consistent with the actual printing process. At any time of printing, the lowest and highest temperatures are distributed on the latest printed unit and gradually dispersed around. In addition, two nodes were randomly selected to compare the temperature variation, as shown in Figure 34a. From Figure 34b,c, it is observed that the temperature curves of both nodes have two evident peaks, which were caused by the high-temperature nozzle depositing materials next to the nodes. 

**Figure 35:**
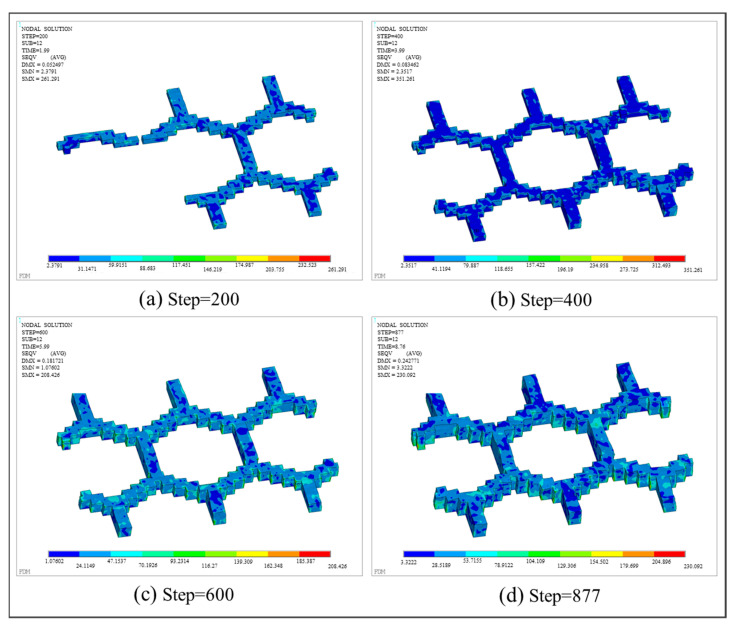
Stress nephogram of the honeycomb structure at different load steps.

**Figure 36:**
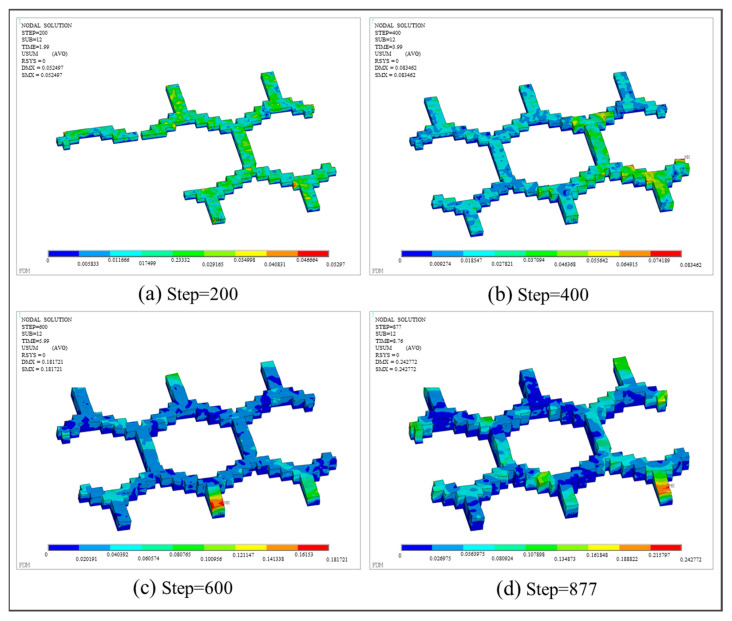
Displacement nephogram of the honeycomb structure at different load steps.

**Figure 37:**
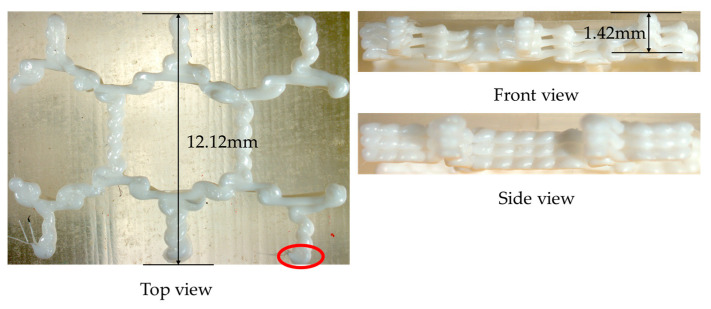
Three views of the real printed honeycomb structure.

With respect to the thermo-mechanical coupling analysis, the distributions of equivalent stress and displacement are shown in Figure 35 and Figure 36, respectively. In the honeycomb structure, the maximum equivalent stress is mainly distributed at the step junctions. Due to the small dimensions of the part, the nozzle passed through the step junctions multiple times during the printing process, preventing effective stress release. Moreover, the deformation accumulated layer by layer with the increase in the layer height, resulting in the maximum deformation concentrated at the last printed layer. Compared with the real printed structure, it can be seen from Figure 37 that due to the small size of the parts and the high thickness of each layer, there is obvious delamination at the junction of each layer, and the warpage deformation at the edge of the grid shows a trend of layer-by-layer accumulation.

Similar to the deformation trend of the previous two numerical examples, the model exhibits small deformation within the X-Y plane, as shown in the top view and the side view. In Figure 36d, the maximum deformation is 0.242772 mm, which is located at the edge of the top surface, as marked by the red circle in Figure 37. A vernier caliper measurement at this position reveals that the total thickness of the printed part is 1.42 mm, whereas the thickness of the CAD model is 1.2 mm. Thus, the deformation of the printed part is 0.15 mm, and the relative error relative to the theoretical value is 9.38%. The result validates the consistency between the simulation result and the real printed result. It also proves that the proposed path-based discrete modeling and process simulation method is fully applicable to the FDM simulation of complex structures.

## 4. Conclusions

In this work, a path-based discrete modeling and process simulation method was proposed for the FDM process. Starting from the CAM stage, the process parameters and material deposition toolpaths were sufficiently utilized to reconstruct the FE model. Then, the BDEM was applied to simulate the FDM process. On this basis, the indirect coupling method was adopted to solve the thermo-mechanical coupling field. Finally, the distributions of temperature, stress, and displacement were obtained to predict the possible warping deformation. The environment was set up in ANSYS APDL and the corresponding computing programs were compiled in C++. The numerical examples, conducted using ABS filaments, effectively validate that the simulated deformations align with those of real printed parts. The proposed method thoroughly considers the impacts of process parameter settings and path planning on FDM structures, which enables the rapid simulation of complex geometries under diverse process parameters. Moreover, it is applicable to other mainstream AM processes, without being constrained by complex geometries. The deformation forecasts derived from the simulation model can offer valuable guidance for both structural optimization and the adjustment of process parameters.

This study was mainly centered on the process simulation of FDM. Owing to limitations in computing resources, only small-scale structures were analyzed through numerical examples. Future work will concentrate on the simulation of other AM processes for large-scale structures. The adopted BDEM will be further improved to reduce the influence of non-activated elements on the thermo-mechanical coupling analysis. Additionally, the flow field will be introduced based on current research to improve the accuracy of the deformation prediction.

## Figures and Tables

**Table 1:** G-code commands for machine operation.

Machine Operations	G-Code Commands	Meanings	Parameters	Meanings
Linear nozzle movement	G0	Linear movement without material extrusion	F	Material feeding rate (mm/min)
	G1	Linear movement with material extrusion	X	Target coordinate on the X-axis
Nozzle heating	M104	Start heating the nozzle to the target temperature	Y	Target coordinate on the Y-axis
	M109	Wait for the nozzle to heat to the target temperature	Z	Target coordinate on Z-axis
Hot bed heating	M140	Start the hot bed heating to the target temperature	E	Length of material extrusion per toolpath (mm)
	M190	Wait for the hot bed heating to reach the target temperature	S	Target temperature (°C)

**Table 2:** Software and hardware setup.

Software/Hardware	Configurations
Slicing software	Ultimaker Cura 5.2.1
FE analysis software	Mechanical APDL Product Launcher 2022 R1
Programming software	Microsoft Visual Studio 2008 (language: C++)
FDM material	Acrylonitrile-butadiene-styrene (ABS)
FDM machine	Bambu Lab X1-Carbon model 3D printer
Nozzle diameter	0.4 mm

**Table 3:** ABS material properties.

Temperature (°C)	Thermal Conductivity	Specific Heat Capacity (J)	Young’s Modulus	Poisson’s Ratio	Density (Kg/m^3^)	Coefficient of Thermal Expansion
50	0.03	1470	3.50 × 10^5^	0.38	1150	8.51 × 10^−5^
100	0.028	1490	2.48 × 10^9^	0.39	1150	8.42 × 10^−5^
150	0.029	1710	1.68 × 10^9^	0.40	1150	8.40 × 10^−5^
250	0.033	2020	0.50 × 10^9^	0.41	1150	8.38 × 10^−5^

**Table 4:** Major process parameters for FE simulation and FDM printing.

Process Parameters	Values	Units
Nozzle temperature	210	°C
Hot bed temperature	50	°C
Printing speed	40	mm/s
Layer thickness	0.4	mm

## Data Availability

The original contributions presented in this study are included in the article. Further inquiries can be directed to the corresponding author.
